# The V Protein of Canine Distemper Virus Is Required for Virus Replication in Human Epithelial Cells

**DOI:** 10.1371/journal.pone.0082343

**Published:** 2013-12-17

**Authors:** Noriyuki Otsuki, Yuichiro Nakatsu, Toru Kubota, Tsuyoshi Sekizuka, Fumio Seki, Kouji Sakai, Makoto Kuroda, Ryoji Yamaguchi, Makoto Takeda

**Affiliations:** 1 Department of Virology 3, National Institute of Infectious Diseases, Musashimurayama, Tokyo, Japan; 2 Laboratory of Bacterial Genomics, Pathogen Genomics Center, National Institute of Infectious Diseases, Shinjyuku, Tokyo, Japan; 3 Department of Veterinary Pathology, Faculty of Agriculture, University of Miyazaki, Miyazaki, Miyazaki, Japan; Lovelace Respiratory Research Institute, United States of America

## Abstract

Canine distemper virus (CDV) becomes able to use human receptors through a single amino acid substitution in the H protein. In addition, CDV strains possessing an intact C protein replicate well in human epithelial H358 cells. The present study showed that CDV strain 007Lm, which was isolated from lymph node tissue of a dog with distemper, failed to replicate in H358 cells, although it possessed an intact C protein. Sequence analyses suggested that a cysteine-to-tyrosine substitution at position 267 of the V protein caused this growth defect. Analyses using H358 cells constitutively expressing the CDV V protein showed that the V protein with a cysteine, but not that with a tyrosine, at this position effectively blocked the interferon-stimulated signal transduction pathway, and supported virus replication of 007Lm in H358 cells. Thus, the V protein as well as the C protein appears to be functional and essential for CDV replication in human epithelial cells.

## Introduction

The genus *Morbillivirus* in the family *Paramyxoviridae* consists of six members: measles virus (MV), rinderpest virus, phocine distemper virus, peste-des-petits-ruminants virus, cetacean morbillivirus, and canine distemper virus (CDV). These viruses cause acute systemic infections in specific animal species [1]. Among them, CDV primarily infects dogs and other canidae, causing distemper characterized by fever, coughing, vomiting, diarrhea, and neurological manifestations [2]. CDV also infects many other animal species [3]–[10]. Most importantly, a great number of non-human primates have been lethally infected by CDV in the last several years [11]–[13]. Consequently, it is of concern whether CDV possesses or can acquire the ability to infect humans.

Viruses in the genus *Morbillivirus* are enveloped and possess a nonsegmented negative-stranded RNA genome encoding six genes, N, P/V/C, M, F, H, and L [1]. The nucleocapsid (N) protein encapsidates the virus genome, forming a ribonucleocapsid with helical symmetry. The viral RNA-dependent RNA polymerase, which is composed of the phospho- (P) and large (L) proteins, binds to the ribonucleocapsid, and transcribes and replicates the virus genome in infected cells. In addition to the P protein, two nonstructural proteins, V and C, are encoded by the P gene by a process of RNA editing and an alternative translation initiation in a different reading frame, respectively [1]. The V and C proteins are nonessential products for virus replication, but critical for counteracting the host innate immune responses [14]–[17]. Virus particles possess two types of transmembrane glycoproteins, the hemagglutinin (H) and fusion (F) proteins, on the envelope. The H protein binds to receptors on host cells, and the F protein mediates membrane fusion between the viral envelope and the host cell plasma membrane.

Previous studies have demonstrated that signaling lymphocytic activation molecule (SLAM) and nectin-4 are common receptors for CDV and MV [18]–[22]. SLAM is expressed on cells of the immune system, and nectin-4 is expressed in the epithelial cells of various organs [23]. Nectin-4 is exclusively expressed at the basolateral surface of differentiated epithelial cells, and thus epithelial cells can only be infected by immune cells infiltrating the epithelial submucosa [24], [25]. Although the abilities to use SLAM and nectin-4 are shared by MV and CDV, CDV preferentially uses dog SLAM (dSLAM) [18], [26] and shows a disability in using human SLAM (hSLAM) [13], [27]–[30]. However, a single amino acid change in the H protein is sufficient for CDV to adapt to use hSLAM as a receptor [27]–[29]. Conversely, CDV is intrinsically able to use human nectin-4 (hNectin4) as well as dog nectin-4 (dNectin4) as a receptor [30]. Thus, CDV easily adapts to use both human immune and epithelial cell receptors [28]–[30]. Nonetheless, MV antigens can induce cross-reactive cellular and humoral immunity against CDV [31]–[33]. Hence, CDV is unlikely to adapt to humans in the presence of immunity to MV, but could do so if MV was eradicated [34].

In our previous study, we demonstrated that wild-type (wt) CDV strains have the potential to replicate in human epithelial H358 cells by using hNectin4 as a receptor [30]. H358 cells are derived from the human lung, and highly permissive to MV infection [35]. Although nectin-4 is expressed at the basolateral surface of epithelia, H358 cells can be infected with MV from either side because they form incomplete tight junctions. In the present study, the term “wt CDV strains” is used for CDV strains isolated directly from dogs with distemper using Vero cells constitutively expressing dSLAM (Vero.DogSLAMtag) [18], [36]–[38]. Our previous study demonstrated that the C protein of CDV is functional in human cells and sufficiently supports CDV replication in these cells [30]. However, we recently identified a wt CDV strain, 007Lm, that fails to replicate in H358 cells, although it possesses an intact C protein. Through analyses of the 007Lm strain, we identify another factor required for CDV to ensure its replication in human epithelial cells.

## Materials and Methods

### Cells and viruses

H358 cells [35] were maintained in RPMI medium supplemented with 10% fetal bovine serum (FBS). The cDNAs encoding the V-Cys_267_ and V-Tyr_267_ proteins were inserted into the pcDNA3.1(-)-V5 vector possessing a V5 tag sequence, generating pcDNA3.1(-)-V-Cys_267_ and pcDNA3.1(-)-V-Tyr_267_. H358 cell clones stably expressing the V-Cys_267_ and V-Tyr_267_ proteins (H358-V-Cys_267_-5, H358-V-Cys_267_-6, and H358-V-Tyr_267_-11) were generated by transfecting H358 cells with pcDNA3.1(-)-V-Cys_267_ and pcDNA3.1(-)-V-Tyr_267_, respectively. H358-V-Cys_267_-5, H358-V-Cys_267_-6, and H358-V-Tyr_267_-11 cells were maintained in RPMI medium supplemented with 10% FBS and 1 mg/ml geneticin (G418; Invitrogen Life Technologies, Carlsbad, CA). Vero.DogSLAMtag and Vero/hSLAM cells [18], [39] were maintained in DMEM supplemented with 5% FBS and 1 mg/ml geneticin. 007Lm-VDS was isolated from lymph node tissue obtained from a dog with distemper using Vero.DogSLAMtag cells [18] and passaged 5–10 times in these cells [38]. 007Lm-H358p8 was obtained as follows. 007Lm-VDS was inoculated into monolayers of H358 cells and passaged eight times in these cells. After the eight passages, the infected cells were scraped into culture medium, subjected to three cycles of freezing-and-thawing, and centrifuged. The supernatant was collected. MVΔV, which expresses EGFP and lacks V protein expression, was reported previously [40].

### Replication kinetics

Monolayers of H358-V-Tyr_267_-11, H358-V-Cys_267_-6, and parental H358 cells in 24-well plates were infected with 007Lm-VDS or 007Lm-H358p8 at a MOI of 0.01 per cell. After various time intervals, the cells were scraped into the culture supernatants, and the virus titers (plaque-forming units: PFU) were determined by plaque assays in Vero.DogSLAMtag cells.

### Virus titration

For CDV, monolayers of Vero.DogSLAMtag cells in 6-well cluster plates were infected with serially diluted virus samples, incubated for 1 h at 37°C, and overlaid with 3 ml of minimal essential medium containing 2% FBS and 0.8% agarose (0.8% agarose-MEM). At 7 days post-infection (p.i.), 2 ml of 0.8% agarose-MEM containing 0.01% neutral red was overlaid on each well, and the PFU was determined by counting the number of plaques at 2 days after the procedure. For MVΔV, which expresses EGFP [40], monolayers of Vero/hSLAM cells on 24-well cluster plates were infected with serially diluted virus samples, incubated for 1 h at 37°C, and overlaid with 1 ml of 0.8% agarose-MEM. At 48 h p.i., the numbers of EGFP-expressing cell foci were counted under a fluorescence microscope. The numbers were expressed as focus-forming units (FFU).

### Next-generation sequencing

Culture supernatants of Vero.DogSLAMtag and H358 cells infected with 007Lm-VDS and 007Lm-H358p8, respectively, were collected, and the virus particles were centrifuged into pellets through 30% sucrose cushions (30% sucrose [wt/vol] in NTE [0.1 M NaCl, 0.01 M Tris pH 7.4, 0.001 M EDTA]) at 185,000×*g* for 2 h in a Beckman 45 Ti rotor at 4°C. Subsequently, viral RNA was purified from the virus particles using Isogen (Nippon Gene, Tokyo, Japan). An RNAseq library was prepared using a ScriptSeq™ v2 RNA-Seq Library Preparation Kit (Illumina-compatible) (Epicentre Biotechnologies, Madison, WI) and the indexing method. Deep sequencing runs for pair-end short reads were performed with MiSeq and GAIIx systems (Illumina, San Diego, CA). To identify nucleotide variations based on the RNAseq analysis, the obtained short reads were mapped to the corresponding reference CDV genome sequence (007Lm-VDS) by the BWA mapping tool [41]. The obtained mapping data were visualized with GenomeJack viewer software (Mitsubishi Space Software, Tokyo, Japan).

### Direct sequencing of the C protein-coding region

Viral RNAs were extracted from each virus stock using a High Pure Viral RNA Kit (Roche Diagnostics GmbH, Mannheim, Germany) according to the manufacturer's instructions. First-strand cDNA was synthesized using Super Script III reverse transcriptase (Invitrogen, Carlsbad, CA), and then amplified by PCR using Prime STAR GXL DNA polymerase (Takara Bio, Shiga, Japan). The primers used for amplification and sequencing are available upon request. The PCR products were purified using a QIAquick Gel Extraction Kit (Qiagen KK, Tokyo, Japan). The nucleotide sequences of the purified PCR products were determined using a Big Dye Terminator v3.1 Cycle Sequencing Kit (Applied Biosystems, Foster City, CA) and a capillary sequencer.

### Nucleotide sequence accession numbers

The short reads obtained by the next-generation sequencing have been deposited in the DDBJ Sequence Read Archive (DRA) of Japan (accession number: DRA000586). The consensus genome nucleotide sequences of 007Lm-VDS and 007Lm-H358p8 have been deposited in DDBJ/GenBank with accession numbers AB823706 and AB823707, respectively.

### Immunoblotting

Monolayers of H358-V-Tyr_267_-11, H358-V-Cys_267_-5, H358-V-Cys_267_-6, and parental H358 cells were lysed in RIPA buffer (150 mM NaCl, 10 mM Tris-HCl pH 7.4, 1% Triton X-100, 1% sodium deoxycholate, 0.1% SDS). Polypeptides in the cell lysates were separated by SDS-PAGE, and electroblotted onto nitrocellulose membranes (Hybond-ECL; Amersham Biosciences, Amersham, UK). Subsequently, the membranes were incubated with anti-V5 (Invitrogen) or anti-tubulin (clone B-5-1-2; Sigma, St. Louis, MO) antibodies, followed by incubation with a peroxidase-conjugated secondary antibody. The antibody-bound peptides were detected using an ECL Advance Western Blotting Kit (GE Healthcare, Little Chalfont, UK). The chemiluminescent signals were detected and visualized using an LAS 1000plus Image analyzer (Fuji Film, Tokyo, Japan).

### Immunofluorescence and confocal microscopy

007Lm-VDS-infected cells were cultured for 48 h, fixed with phosphate-buffered saline (PBS) containing 4% paraformaldehyde, and permeabilized with PBS containing 0.5% Triton X-100. After washing with PBS, the cells were incubated with an antiserum for CDV and an anti-human IRF-3 antibody (BD Biosciences, San Diego, CA), followed by incubation with secondary antibodies conjugated with Alexa Fluor 488 and 594, respectively. For counterstaining of the nuclei, the cells were incubated with DAPI. After the fluorescence staining, the cells were observed using an FV1000D Spectral Type Confocal Laser-scanning Microscope (Inverted Microscope IX81; Olympus, Tokyo, Japan). Data analyses were performed using FV10-ASW (Olympus) and ImageJ (National Institutes of Health, Bethesda, MD) software.

### Quantification of transcripts of interferon-stimulated genes (ISGs) by RT-qPCR

Monolayers of H358-V-Tyr_267_-11, H358-V-Cys_267_-6, and parental H358 cells were treated with 100 U/ml of IFN-β for 0, 2, 4, and 8 h. Total RNA was extracted using Trizol reagent (Invitrogen) and cDNA was synthesized with a Transcriptor First-strand cDNA Synthesis Kit (Roche Applied Science, Indianapolis, IN) using random hexamers. Quantitative PCR was performed in a LightCycler 480 Instrument (Roche Applied Science) with a universal probe library according to the manufacturer's procedure. The relative amounts of cDNAs for ISG15, OAS1, and ISG56 were calculated by normalization with GAPDH or HPRT1.

## Results

### Growth defect of wt CDV 007Lm-VDS in human epithelial H358 cells and its adaptation to growth in these cells

**Figure 1:**
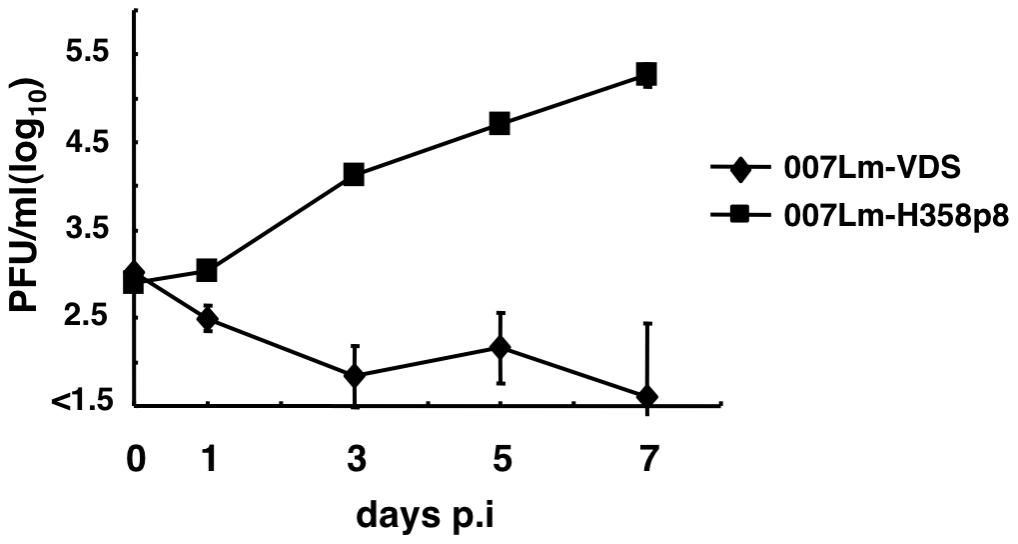
Replication kinetics in H358 cells. H358 cells were infected with 007Lm-VDS or 007Lm-H358p8 at a MOI of 0.01. At 1, 3, 5, and 7 days p.i., the virus titers were determined by plaque assays. Data represent the means ± standard deviations of the results from triplicate samples.

Our previous paper demonstrated that wt CDV strains possessing an intact C protein (Ac96I-H358, 82Con, 55L, M24Cr, and Th12) replicate well in H358 cells [30]. However, during analyses of other wt CDV strains, we found that wt CDV 007Lm [38] failed to replicate in H358 cells, despite the fact that it possesses an intact C protein. The 007Lm strain was directly isolated from lymph node tissue obtained from a dog with distemper using Vero.DogSLAMtag cells [18] and passaged several times in these cells [38]. Dogs infected with the 007Lm strain showed clinical signs: diarrhea with blood, ocular discharge, depression, soft face, and high fever [38]. Lymphopenia was also evident in these animals [38]. In the present study, this Vero.DogSLAMtag cell-isolated 007Lm strain was termed “007Lm-VDS”. Since RNA viruses form quasispecies, 007Lm-VDS was considered to be a mixed population, rather than a single clone, as previously discussed for Ac96I-VDS [30]. H358 cells were infected with 007Lm-VDS, and the virus titers were determined at 1, 3, 5, and 7 days p.i. The data showed that 007Lm-VDS replicated at minimum levels in H358 cells (Fig. 1). Nucleotide sequence analyses of the C protein-coding region by direct sequencing assays using RT-PCR products as templates suggested that, unlike Ac96I-VDS, 007Lm-VDS possessed an intact C protein, similar to other wt strains (82Con, 55L, M24Cr, Th12, and Ac96I-H358). To obtain H358 cell-adapted 007Lm, 007Lm-VDS was passaged eight times in H358 cells. After the eight passages, 007Lm-VDS became able to cause extensive syncytium formation in H358 cells (data not shown). This 007Lm population after eight passages in H358 cells was termed “007Lm-H358p8” in the present study. H358 cells were infected with 007Lm-H358p8, and the infectious virus titers were analyzed at 1, 3, 5, and 7 days p.i. Unlike 007Lm-VDS, 007Lm-H358p8 replicated efficiently in H358 cells (Fig. 1). These data demonstrate that 007Lm-VDS with an intact C protein failed to replicate in H358 cells, but readily adapted to grow in these cells.

### Genetic changes in CDV 007Lm-H358p8 during propagation in H358 cells

As performed for the Ac96I strains in our previous study [30], the nucleotide sequences of 007Lm-VDS and 007Lm-H358p8 were analyzed by next-generation sequencing. A list of the nucleotide substitutions acquired by 007Lm-H358p8, as well as selected preexisting nucleotides, during the eight passages of 007Lm-H358p8 in H358 cells are shown in Table 1. As reported previously [30], the list shows all nucleotide positions where more than 15% of the 007Lm-H358p8 genome acquired a nucleotide substitution or the proportion of preexisting nucleotides was increased by more than 15%. Among them, three nucleotide substitutions were predicted to cause amino acid changes. The first one was located at nucleotide position 2599. Almost half (48%) of the 007Lm-VDS genome reads possessed an adenine at the position, and the remaining reads (52%) possessed a guanine (Table 1). Since the region containing nucleotide position 2599 encodes both the P and V proteins, the nucleotide change was predicted to cause amino acid changes in both the P and V proteins. The P protein encoded in the genome with the adenine and the guanine possessed a methionine and a valine, respectively (Table 1). Importantly, the genome with the adenine encoded a V protein possessing a tyrosine (Tyr_267_), instead of a cysteine (Cys_267_), at amino acid position 267 (Table 1). Cys_267_ is one of the highly conserved cysteine residues among the V proteins of *Paramyxovirus* family members [42], and is required for the formation of a zinc-binding fold [43]. The majority (97%) of 007Lm-H358p8 possessed a guanine, rather than an adenine, at nucleotide position 2599, and thus the V protein possessed the conserved Cys_267_. The second mutation was predicted in the F protein, through a nucleotide substitution at position 5281 (Table 1). The third mutation was at nucleotide position 8721, and caused an amino acid change in the H protein. The sequence data suggested that approximately half (55%) of 007Lm-VDS possessed a threonine at amino acid position 548 of the H protein, while the remainder (45%) possessed a methionine at that position (Table 1). After eight passages in H358 cells, the population possessing a methionine was selected (100% of 007Lm-H358 possessed a methionine at that position in the H protein). These data suggest that the efficient replication after eight passages was not necessarily induced by V protein mutations, but could also be influenced by H protein mutations.

**Table 1:** Acquired and selected nucleotides during passages in NCI-H358 cells.

		007Lm-VDS					007Lm-H358p8						
			Proportion (%)					Proportion (%)					
Gene	Nucleotide position	Total no. of reads	A	C	G	T	Total no. of reads	A	C	G	T	ORF	Amino acid substitution
P/V	2599	5725	48	0	52	0	1155	3	0	97 a	0	P	M267V
												V	Y267C
F	5281	171	1	0	99	0	56	76	0	23	0	F	C116Y
F	6885	583	0	9	0	90	459	0	76	0	24	F	no
H	8721	42	0	52	0	48	72	0	100	0	0	H	M548T
L	13079	1760	46	0	53	0	440	3	0	97	0	L	no
L	14939	1251	89	0	11	0	467	12	0	88	0	L	no

^a^ Acquired and selected nucleotides are underlined.

### Functions of CDV V proteins with Tyr_267_ and Cys_267_ in supporting the growth of CDV 007Lm-VDS and V protein-deficient MV in H358 cells

**Figure 2:**
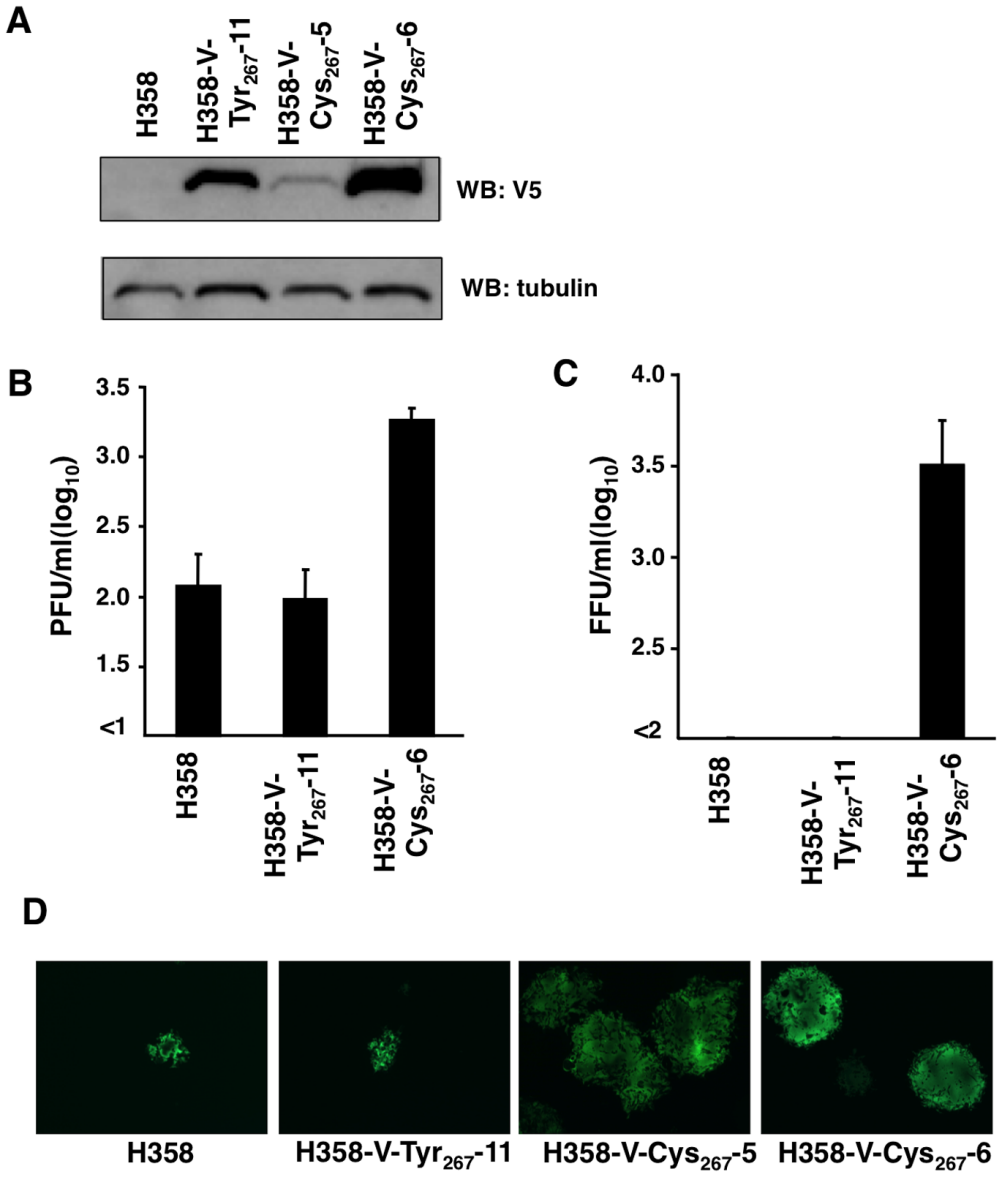
Virus growth in the parental and V protein-expressing H358 cells. (A) Immunoblotting. The parental H358, H358-V-Tyr_267_-11, H358-V-Cys_267_-5, and H358-V-Cys_267_-6 cells were lysed in RIPA buffer, and subjected to SDS-PAGE followed by immunoblotting for detection of the V protein. Tubulin was detected as an internal control. (B, C) The parental H358, H358-V-Tyr_267_-11, and H358-V-Cys_267_-6 cells were infected with 007Lm-VDS (B) or MVΔV (C) at a MOI of 0.01. At 5 days p.i., the virus titers (PFUs and FFUs, respectively) were determined. (D) Observation of EGFP-expressing MVΔV-infected cells (syncytia) using a fluorescence microscope.

Because our previous paper demonstrated that the H and F proteins of wt CDV strains are intrinsically functional in H358 cells [30], we focused on analyses of the V proteins. To assess the functions of the V proteins with a tyrosine and a cysteine at amino acid position 267 (V-Tyr_267_ and V-Cys_267_, respectively), these proteins were constitutively expressed in H358 cells. H358 cells expressing V-Tyr_267_ were termed “H358-V-Tyr_267_-11”, and those expressing V-Cys_267_ were termed “H358-V-Cys_267_-6” (Fig. 2A). Another H358 cell clone, H358-V-Cys_267_-5, expressing a low level of V-Cys_267_ was also generated (Fig. 2A). H358-V-Tyr_267_-11, H358-V-Cys_267_-6, and parental H358 cells were infected with 007Lm-VDS at a MOI of 0.01, and the infectious virus titers were determined at 5 days p.i. (Fig. 2B). In H358-V-Cys_267_-6 cells, 007Lm-VDS produced ∼10 times greater amounts of infectious virus particles compared with H358-V-Tyr_267_-11 and parental H358 cells (Fig. 2B). The virus titers, albeit low, in H358-V-Tyr_267_-11 and parental H358 cells were comparable to one another. These data suggest that the V-Cys_267_ protein, but not the V-Tyr_267_ protein, had the ability to promote 007Lm-VDS replication in H358 cells. The data further suggest that the growth defect of 007Lm-VDS in H358 cells was at least partly caused by the dysfunction of V-Tyr_267_. We also examined whether V-Cys_267_ could compensate for the growth defect of V protein-deficient MV (MVΔV) [40] in H358 cells. MVΔV failed to spread and produced infectious virus particles at minimum levels in H358-V-Tyr_267_-11 and parental H358 cells (Fig. 2C, 2D). Conversely, MVΔV spread well and produced infectious virus particles in H358-V-Cys_267_-6 cells (Fig. 2C, 2D). Even in H358-V-Cys_267_-5 cells, where only a low level of V-Cys_267_ was expressed, 007Lm-VDS spread as efficiently as in H358-V-Cys_267_-6 cells, forming large syncytia (Fig. 2D). These data demonstrate that the CDV V-Cys_267_ protein was fully functional, similar to the MV V protein, in H358 cells.

### Interference with IFN-induced signal transduction by the V-Cys_267_ protein in H358 cells

**Figure 3:**
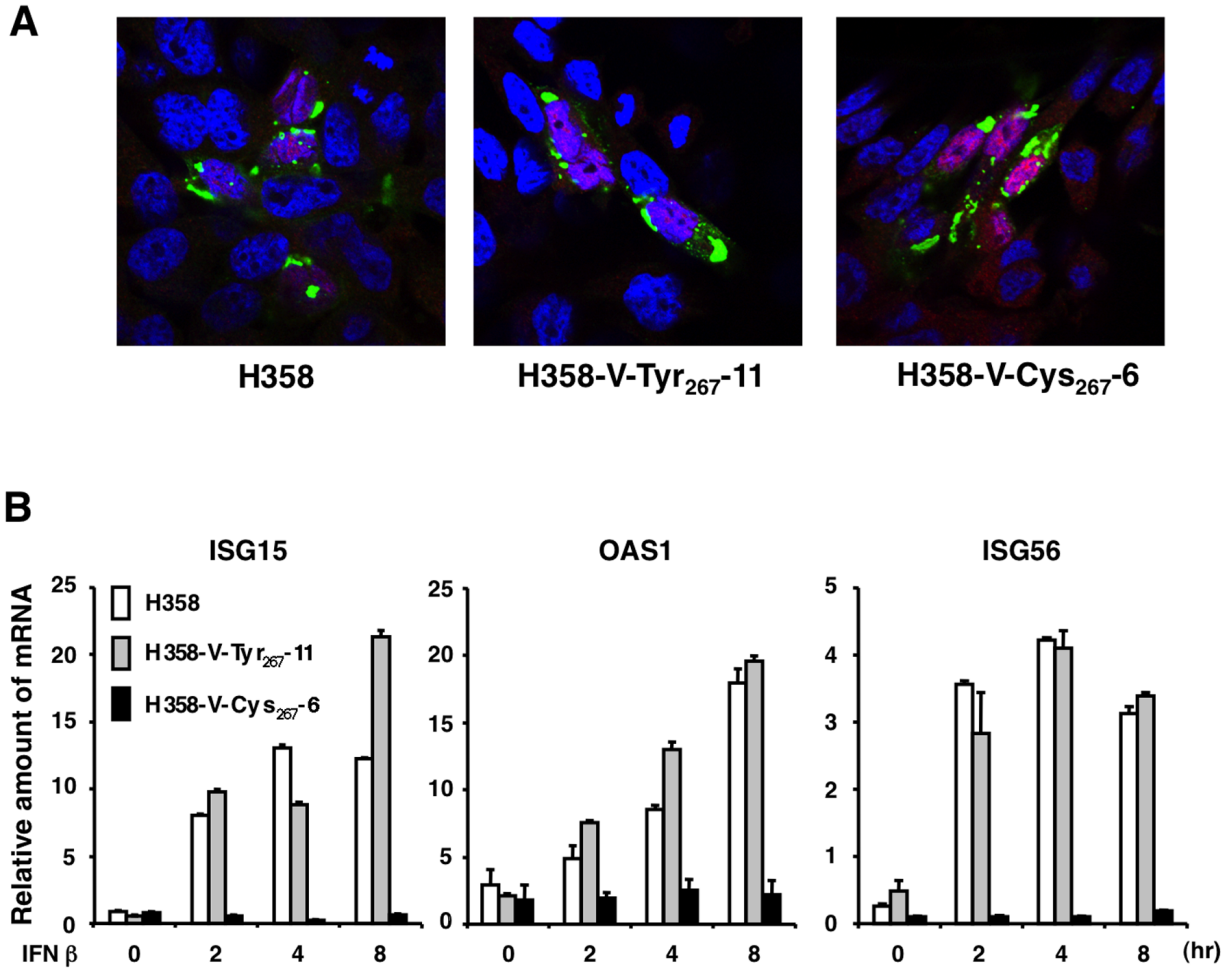
IFN-related responses in the parental and V protein-expressing H358 cells. (A) The parental H358, H358-V-Tyr_267_-11, and H358-V-Cys_267_-6 cells were infected with 007Lm-VDS, and CDV antigens (green) and IRF-3 (red) were detected by immunofluorescence confocal microscopy. The nuclei (blue) were detected by counterstaining with DAPI. (B) The parental H358, H358-V-Tyr_267_-11, and H358-V-Cys_267_-6 cells were untreated (0 h) or treated with 100 U/ml of IFN-β for 2, 4, and 8 h. Total RNAs were purified and the relative amounts of mRNAs for three ISGs (ISG15, OAS1, and ISG56) were measured by RT-qPCR.

Host cells detect RNA virus infections using specific intracellular sensor molecules, RIG-I and mda-5 [44], [45]. These sensor molecules transmit signals to a downstream adapter molecule, which in turn activates specific kinases [46]. The activated kinases phosphorylate IFN-regulatory factors (IRFs), including IRF3, which is translocated into the nucleus, leading to the production of IFN. Previous studies have indicated that negative-strand RNA virus infection is recognized by RIG-I, but not by mda-5 [47], [48]. For MV, the V protein, but not the C protein, has a function in blocking the IFN induction pathway, but interferes with the mda-5-mediated IFN induction pathway, rather than the RIG-I-mediated pathway [49]. Our previous study showed that H358 cells have a functional IFN system [40]. H358-V-Tyr_267_-11, H358-V-Cys_267_-6, and parental H358 cells were infected with 007Lm-VDS at a MOI of 0.1, and an indirect immunofluorescence assay for detection of IRF3 was performed at 2 days p.i. IRF3 was detected in the cytoplasm of cells uninfected by 007Lm-VDS (Fig. 3A). Conversely, strong IRF3 signals were detected in the nuclei of 007Lm-VDS-infected cells (Fig. 3A). This nuclear translocation of IRF3 was observed in all three cell lines (Fig. 3A). These data suggest that both the V-Tyr_267_ and V-Cys_267_ proteins were incapable of blocking the IFN-induction pathway. Secreted IFN activates the Jak/Stat signaling pathway and stimulates the transcription of a variety of ISGs possessing antiviral functions [50]. H358-V-Tyr_267_-11, H358-V-Cys_267_-6, and parental H358 cells were treated with IFN for 2, 4, and 8 h, and the mRNA levels of three ISGs (ISG15, OAS1, and ISG56) were analyzed by RT-qPCR. The mRNA levels of all three ISGs were elevated in H358-V-Tyr_267_-11 and parental H358 cells in response to IFN treatment (Fig. 3B). Conversely, the ISGs were completely unstimulated in H358-V-Cys_267_-6 cells (Fig. 3B). These data demonstrate that the CDV V-Cys_267_ protein effectively interfered with the signal transduction of the human IFN system.

## Discussion

Dogs and other canidae are the main hosts infected by CDV, but the virus has also caused diseases in many other animal species including non-human primates [3]–[13]. To date, there is no evidence of human infection with CDV. Cross-reactive immunity between MV and CDV may have protected humans against CDV infection [31]–[34]. In addition, CDV uses dog and monkey receptors (both SLAM and nectin-4) very efficiently, but has difficulty in using hSLAM [13], [27]–[30]. Conversely, hNectin4 functions as efficiently as dNectin4 as a CDV receptor [28], [30]. The SLAM incompatibility may be a reason why humans are not infected by CDV. However, it is noteworthy that only a single amino acid change in the H protein is sufficient for CDV to become able to use hSLAM [27]–[29]. Thus, at the receptor level, humans can easily become susceptible to CDV infection. These facts suggest the possibility that CDV may infect humans, causing a measles-like illness in humans in the future. However, the host range of viruses is determined by a variety of factors, and not simply by their receptor usage alone. A critical factor that determines the host range of paramyxoviruses is the ability of each virus to counteract the host IFN system [51]–[54]. In 2013, Iwasaki et al. [54] clearly demonstrated that the IFN-counteracting ability is an important factor that determines the host range of MV.

In our previous paper [30], we demonstrated that most wt CDV strains replicated reasonably well in H358 cells, while an Ac96I isolate failed to replicate in these cells. The study revealed that the growth disability of Ac96I-VDS was caused by a defect in its C protein [30]. These data therefore revealed that the CDV C protein is functional in human cells and critical for CDV replication in these cells [30]. In the present study, we identified another isolate, 007Lm-VDS, with a growth defect in H358 cells. Unlike Ac96I-VDS, 007Lm-VDS possessed an intact C protein, but had a V protein with a mutation at a conserved cysteine residue. Almost half of the 007Lm-VDS population possessed a mutated V protein, in which the conserved Cys_267_ was replaced with Tyr_267_. Most paramyxoviruses encode a V protein with a C-terminal region that contains seven highly conserved cysteine residues [42]. The data for the full-length V protein crystal structure of parainfluenza virus 5 showed a C-terminal globular core domain for the V protein [43]. The core domain is stabilized by a unique zinc-finger motif formed by the seven conserved cysteine residues and a histidine residue [43]. Two large loops coordinate two zinc ions, of which one is coordinated by three conserved cysteine residues and a histidine, and the other is coordinated by four conserved cysteine residues [43]. Cys_267_ is one of the four cysteine residues. Thus, the change from Cys_267_ to Tyr_267_ may disrupt the zinc-finger fold and a core domain structure, thereby losing the V protein functions. Indeed, the IFN-induced signal transduction was completely blocked in H358-V-Cys_267_ cells, while it was unaffected in H358-V-Tyr_267_ cells. These data further demonstrate the importance of the conserved cysteine residues for the paramyxovirus V protein function. Although the V-Cys_267_ protein appeared to be functional, the IFN-induction pathway was not affected, even in H358-V-Cys_267_ cells. These observations would be reasonable, because the V protein does not block the RIG-I-mediated IFN-induction pathway, which is the major pathway against RNA virus infection [47]–[49]. Based on the data for Ac96I-VDS and 007Lm-VDS, we conclude that both the V and C proteins of CDV are sufficiently functional in human epithelial cells to support CDV replication, and that they are critical for CDV replication in these cells.

Both the V and C proteins of CDV have been shown to play important roles for CDV pathogenesis and counteraction of the host IFN responses [14], [55]. However, the respective roles of the CDV V and C proteins in virus pathogenesis remain unclear. These roles are documented for MV. To counteract the host IFN responses, the C and V proteins of MV seem to cooperate mutually by sharing roles with one another [16]. The MV V protein blocks the IFN-induced Jak/Stat signaling pathway [16], [56]–[60]. Although Shaffer et al. [61] reported an inhibitory activity of the MV C protein toward the IFN response, other papers showed that the C protein per se does not have an ability to interfere with the IFN induction pathway [16], [56]–[58]. Instead, the C protein controls the viral RNA polymerase activity [16]. This function of the C protein seems to optimize the level of viral RNA synthesis, indirectly acting to suppress the IFN induction pathway [16]. A host protein, SHCBP1, plays a key role in the C protein function [62]. For MV and CDV, infection via cell-to-cell fusion may be an effective way to counteract the host IFN signal transduction pathway, since viruses can very quickly interfere with this pathway by transferring the accumulated V protein into neighboring cells for infection. However, cooperative actions by the V and C proteins would be critical to effectively evade the host innate immune responses and cause diseases in vivo.

MV antigens induce cross-reactive immunity against CDV [31]–[33], and hSLAM is incompatible with dSLAM for CDV infection. However, it remains unclear which factor is the major barrier for CDV to infect humans. Although a variety of factors may be involved in the determination of virus host range, it is of serious concern that CDV can currently cause diseases in various animal species, including non-human primates, with high mortality [11]–[13]. Our group is now conducting analyses to reveal the molecular bases for how recent CDV strains have acquired the ability to cause severe diseases in non-human primates and which barrier may block CDV transmission to humans.
